# Conflict monitoring with VIIRS Nightfire: the war in Ukraine

**DOI:** 10.1038/s41598-026-42172-0

**Published:** 2026-03-02

**Authors:** Merlijn I. Dingemanse, Mikhail Zhizhin, Daniele Cerra

**Affiliations:** 1 German Aerospace Center (DLR), Earth Observation Center (EOC), Münchener Str. 20, 82234 Weßling, Germany; 2 Payne Institute for Public Policy, Colorado School of Mines, 1500 Illinois St., Golden, CO 80401 USA

**Keywords:** VIIRS, VIIRS Nightfire, Night lights, Ukraine war, Conflict monitoring, Climate sciences, Mathematics and computing

## Abstract

Amid rising geopolitical instability, timely, systematic, and independent monitoring of conflict zones is essential. We show how nighttime thermal anomalies from spaceborne sensors can be combined with boundary data, territorial control vector data, and high-resolution damage assessments to generate indicators of conflict at different levels of analysis. Leveraging the Visible Infrared Imaging Radiometer Suite’s Nightfire product, we extract signals of conflict across Ukraine, tracking the status of heavy industry, delineating the frontline, and detecting urban combat. Although VIIRS data face challenges such as low spatial resolution and susceptibility to atmospheric interference, we find that these can provide valuable insights when paired with supporting geodata. Our results thus support fast, low-cost, and scalable monitoring of conflict zones, enabling timely intelligence in rapidly evolving scenarios.

## Introduction: VIIRS Nightfire—a night watch for Ukraine?

In the era of the *transparent battlefield*^1^, vast amounts of open source geodata from conflict zones are readily accessible. Governance, military, and scientific communities are constantly working to merge, filter, and transform this information into structured pipelines and analytical frameworks, in order to support independent and reliable conflict monitoring. The ongoing Russo-Ukrainian war—the largest and deadliest in Europe since World War II—is a key example. Its scale, intensity, and geopolitical stakes make continuous monitoring essential for understanding the war’s humanitarian, environmental, and strategic impacts. The nighttime data acquired by the Visible Infrared Imaging Radiometer Suite (VIIRS) sensors could make a valuable contribution to conflict monitoring due to the unique combination of high temporal resolution, global coverage, and long timeseries^2^. With three VIIRS sensors in orbit, the revisit time ranges from between three to five visits per night, depending on orbit and latitude. At night, VIIRS can capture not only data in the visible spectrum using its Day-Night-Band (DNB), but also infrared data. The VIIRS Nightfire (VNF)^3–5^ data product uses the sensor’s infrared bands to detect nighttime hot sources such as fires, gas flares, and heavy industry. VNF aggregates all VIIRS overpasses into one file per day with global coverage, allowing for quick and easy accessibility.

VIIRS DNB data has seen extensive use in conflict monitoring: DNB composites were used to show light loss in areas occupied by ISIS during the Syrian civil war^6,7^. With the Russian invasion of Ukraine in February 2022, a number of articles on VIIRS DNB changes in Ukraine were published, where it was extensively shown that most cities showed a significant loss in visible night light^8–11^. On a local scale, it was demonstrated that fires caused by fighting can be detected even in DNB monthly composites by comparing them with very high-resolution (VHR) data^12^. In contrast, the applications of VIIRS infrared emissions for conflict monitoring is not nearly as developed, and mainly focuses on data from the the fire information and resource management system (FIRMS)^13^. For the war in Ukraine, a shift of FIRMS detections towards the frontline was observed, although the studies did not go beyond visual analysis^14,15^. It was also shown that in oblasts affected by fighting, there was a shift in the land cover underneath FIRMS detections away from *agriculture* and towards *urban* and *grassland*^16^. This fact can be seen as an indicator that fires were not caused by regular activities such as the burning of crop remains, but were instead caused by shelling. In another novel usecase, it was demonstrated that there is a correlation between the density of fire detections and *Armed Conflict Location and Event Data*, suggesting the suitability of VIIRS fire detections for monitoring ongoing conflict if shaped correctly^17^.

However, all of this research relies heavily on composite data, limiting VIIRS’s main advantage: high temporal resolution. additionally VNF products are not used, and other relevant data sources related to conflict are not considered. As such, the potential of integrating VNF data with supplementary geodata to generate novel insights into conflicts has not yet been fully explored, a limitation that must be addressed for a potential effective integration into a broader monitoring framework.

**Fig. 1:**
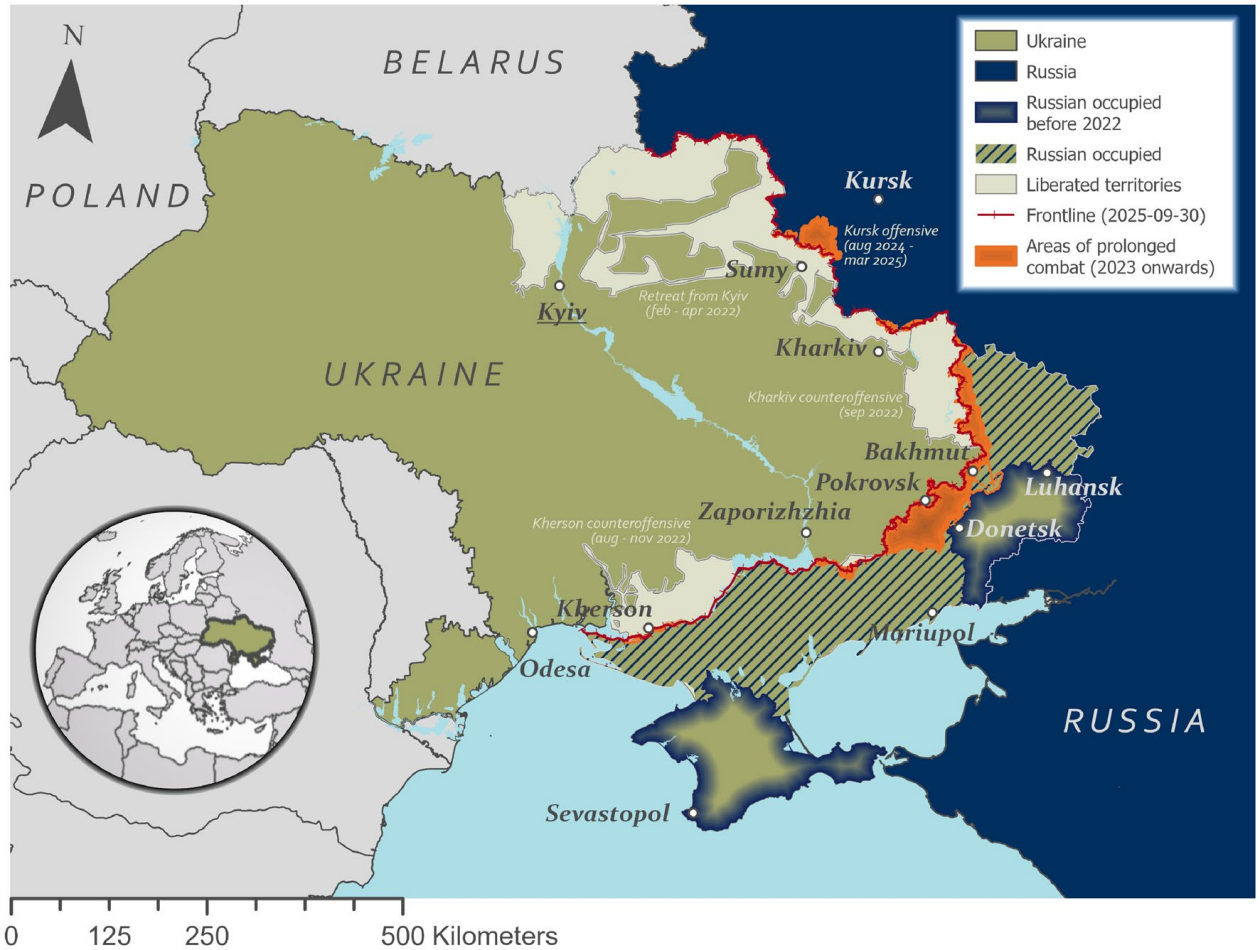
The war in Ukraine as of October 2025, including occupied areas, areas of prolonged combat, the frontline, and relevant towns and cities (Data source: Liveuaumap, ArcGIS living atlas).

This study sets out to address this gap in the form of a capabilities review. Its aim is to demonstrate how the synergy of VNF with other satellite observations and geodata can generate insights on the war in Ukraine at different levels of analysis. Secondary data includes locations of permanent thermal emitters generated from VNF^4^, a variety of boundary data, as well as vector data of area control generated from the *liveuamap* web service^18^. For validation, VNF density was correlated with very high-resolution (VHR) damage detections from the United Nations Satellite Centre (UNOSAT) data base^19^, manually derived by experts. Additionally, selected composites of the optical satellite Sentinel-2 with close acquisition times and limited contamination by clouds were used for visual validation. The area of study is the entirety of Ukraine, and Russian regions bordering Ukraine (see Fig. 1). As the war is still ongoing, a data cut-off date of 2025-09-30 was chosen, allowing for a period of more than 3.5 years to be analysed.

### The war in Ukraine

Since the full-scale Russian invasion in February 2022, the war in Ukraine has unfolded in distinct spatial and temporal phases relevant for remote-sensing-based monitoring (see Fig. 1). The initial phase (February–April 2022) involved a rapid multi-axis Russian advance, including an unsuccessful attempt to seize Kyiv, followed by a withdrawal from northern Ukraine^20^. From spring to summer 2022, fighting concentrated in eastern and southern Ukraine, with Russian forces capturing large parts of Donetsk and Luhansk oblasts, as well as the city of Kherson^21^. Two Ukrainian counteroffensives in autumn 2022 led to the liberation of most of Kharkiv oblast (September 2022) and the west bank of Kherson oblast (November 2022). From early 2023 onward, the frontline largely stabilized along an over 1000 km front, characterized by high-intensity positional fighting and only gradual territorial changes, particularly in Donetsk oblast^20,22^. Across these phases, shifts in frontline position and fighting intensity produced heterogeneous patterns of damage, underscoring the need for monitoring approaches with a high-temporal-resolution.

## Results

### Using VNF to mass monitor urban combat

To assess the humanitarian impacts of the war, focusing on settlements and urban combat is especially valuable, as these areas host the highest concentration of people. Settlements have fixed locations and rarely experience fires except during major incidents, so urban temperature anomalies can serve as an indicator of critical events. Additionally, UNOSAT’s VHR damage database offers a unique opportunity to assess the correlation between urban damage and VNF detections. For several settlements, UNOSAT provides precise counts of damaged buildings, as well as environmental damage such as affected fields and roads, all recorded within selected time windows. Thus, by extracting VNF detections for each settlement within the same time window (excluding those tied to industrial activity), the number of VNF detections can be compared to the number of Comprehensive Damage Assessment (CDA) damage detections. This comparison was computed for all UNOSAT CDA activations in Ukraine not related to flooding (n = 15).

**Fig. 2:**
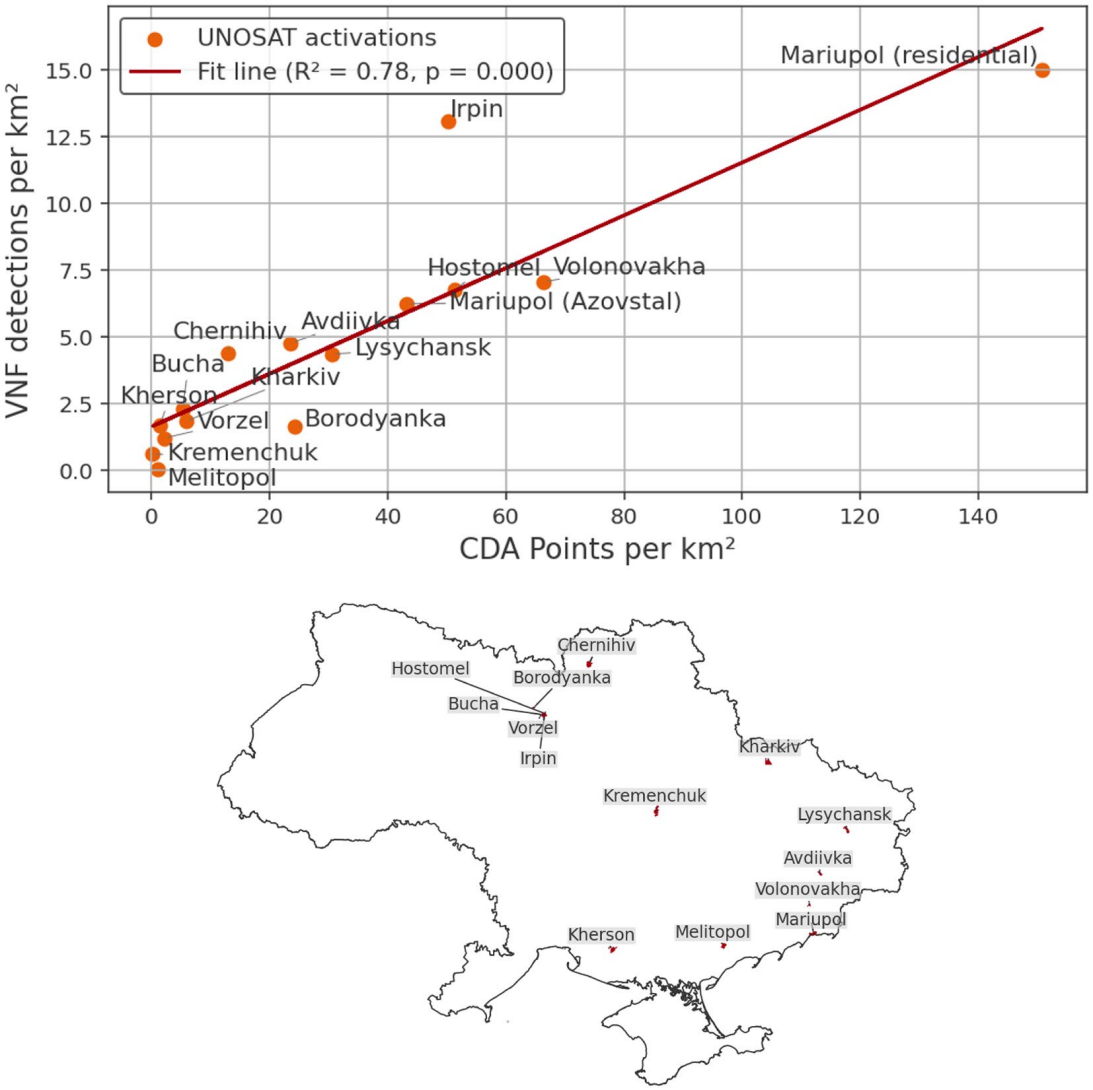
Correlation between density of VNF and CDA (comprehensive damage assessment) detections for settlements with UNOSAT activations in Ukraine. VNF data are selected between the outbreak of the war and the latest CDA post-event VHR image. All CDA damage types are included. The overview map shows areas assessed by UNOSAT (data sources: United Nations Office for the Coordination of Humanitarian Affairs (OCHA), VIIRS Nightfire, UNOSAT).

Figure 2 reveals a statistically significant correlation between the number of VNF detections and the number of CDA damage detections per $\textrm{km}^{2} (\textrm{r}^{2} = 0.78, {p} <0.001),$ with VNF detections increasing with an increasing amount of damage. Correlation is highest when all types of CDA damages are included. The correlation appears to diminish when the overall damage becomes less severe. Nevertheless, in spite of UNOSAT activations coming from the same year and within the same eight months, thus introducing a temporal and seasonal bias in the CDA data, results suggest that VNF can be efficiently used as a proxy to estimate urban damage in Ukraine. These results can be achieved automatically and only relying on open data.

With respect to UNOSAT assessments, analysis based on VNF presents two advantages. First, VNF’s high temporal resolution allows one to estimate an approximate timing for the observed CDA damage. Second, this temporal insight can be extended to any settlement in Ukraine, when integrated with supporting geodata. Such insights would be unfeasible to derive manually, as visual analysis is time-consuming, must be carried out by experts, and relies on the availability of high resolution data. To implement this, VNF detections were extracted for each settlement larger than 5 $\textrm{km}^{2}$ and smoothed using a 7-day rolling window. The area threshold was chosen due to the VIIR’s low spatial resolution making it difficult to attribute VNF detections to even smaller settlements. The 7-day window was chosen to reduce the high day-to-day variability inherent in VNF detections, limiting the impact single events while highlighting sustained combat and associated damage. Then, the weekly distance of each settlement from the frontline was calculated using processed liveuamap data. Next, a list of potentially damaged settlements was generated using empirically determined threshold values. Specifically, we included all settlements that met the following criteria: located within five kilometres of the frontline for at least one week, exhibiting a cumulative total of more than 30 VNF detections, and recording an average density above 2.5 detections per $\textrm{km}^{2}$. These thresholds were set to ensure inclusion of only those settlements with high thermal activity in close proximity to the frontline, thereby reducing the likelihood of false positives. The selected settlements were then labelled as *likely damaged*. For each settlement, the number of VNF detections and the distance from the frontline was then plotted, allowing for quick visual validation. As an additional indicator, the ratio of VNF detections before and after the war was calculated.

**Fig. 3:**
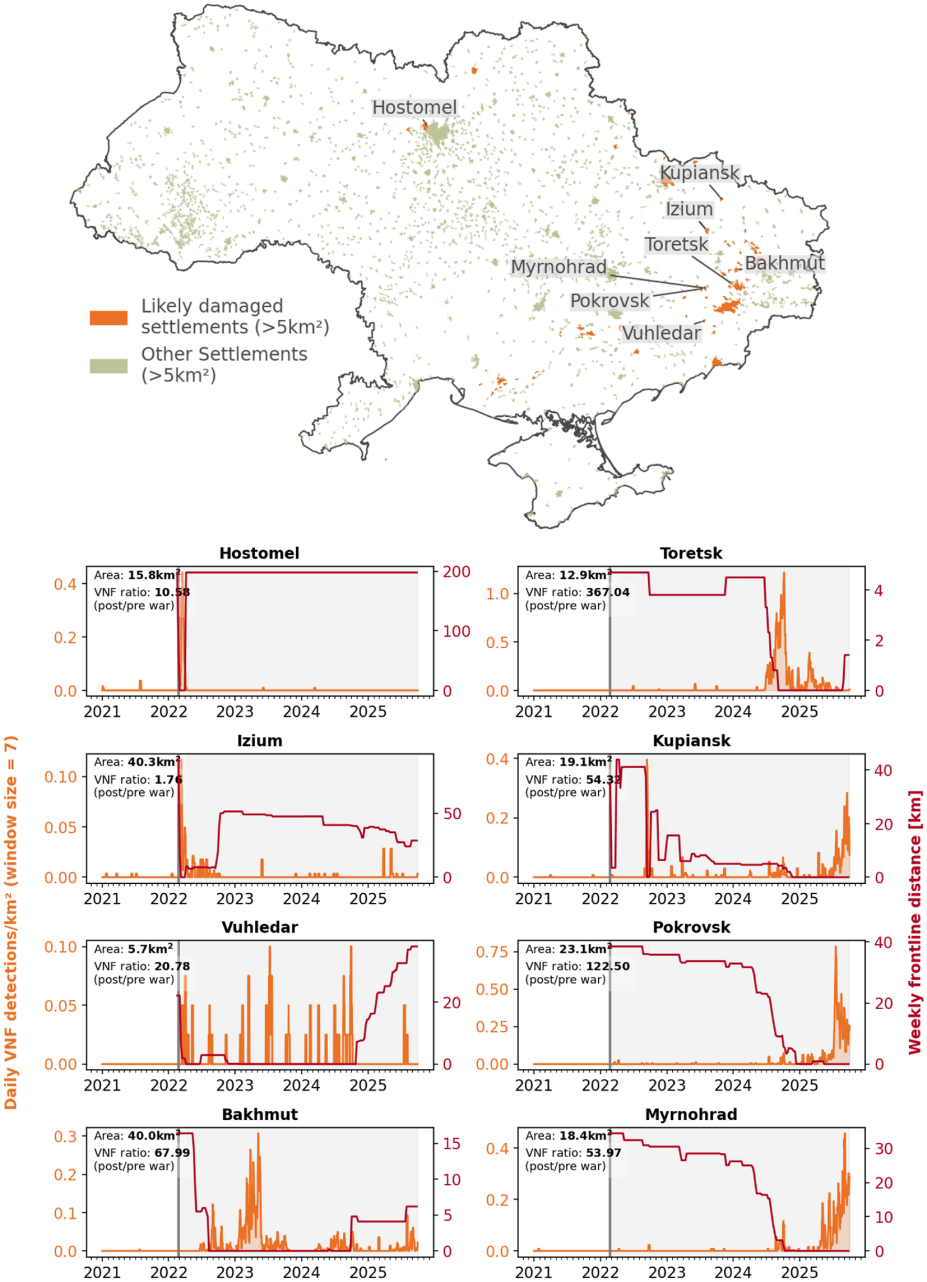
Mass urban combat monitoring. Figure (**a**) shows a spatial overview of Ukrainian settlements with suspected damage identified using VNF detections and a frontline-based thresholding approach. Figure (**b**) presents time series for selected settlements with notable wartime activity. The orange line shows daily VNF detections normalized by settlement area (detections per $\textrm{km}^{2}$), indicating fires, while the red line shows the weekly distance to the active frontline derived from Liveuamap areas of control data (decreasing values indicate the frontline approaching the settlement). Periods where elevated VNF detections coincide with short or rapidly changing frontline distances suggest active urban combat or nearby military operations. For each settlement, the total area ($\textrm{km}^{2}$) and the ratio of post-war (2022–2025) to pre-war (2019–2022) VNF detections are reported. Y-axes are scaled differently to focus on temporal co-variation rather than absolute magnitude. Similar plots can be generated automatically for any Ukrainian settlement (data sources: OCHA, VIIRS Nightfire, Liveuamap).

Following this method, 90 out of the Ukrainian settlements in 1985 larger than 5 $\textrm{km}^{2}$ were labelled as *likely damaged* (see Fig. 3a). Results not reported here are available from the authors on request. Visual inspection of the plots (performed separately by all authors with blind majority voting) shows that 86 percent of these settlements experience an increase in VNF detections as the distance to the frontline decreases. Eight percent show no clear pattern, while six percent show an inverse pattern, with a loss of VNF detections as the distance to the front decreases. Most of the latter cases correspond to settlements with industrial hot sources not listed in the VNF permanent emitter database. Therefore, in these settlements, permanent emitters are dominating the pre-war signal. Settlements where heavy fighting is known to have taken place, such as Hostomel, Bakhmut, and Pokrovsk, all follow the pattern of increased detections (see Fig. 3b). Even very small towns such as Vuhledar show a clearly detectable shift in VNF patterns as the war begins. While several large Ukrainian cities such as Kyiv and Odesa were extensively targeted, the VNF density is not high enough to pass the threshold.

While these results are very promising, the method could benefit from several improvements. First, not all marked settlements show noticeable shifts in heat radiation patterns, especially those with an associated low number of total VNF detections, or located in areas of the Donbas oblast occupied by Russia before 2022. These settlements are characterized by a VNF ratio close to 1. However, filtering using a VNF ratio threshold also removes smaller settlements that do show an increase in VNF detections after the war begins, due to the occasional presence of detections before 2022. More importantly, it is not known how many settlements were excluded that *would* show increases in emissions due to our empirically selected thresholds. This suggests that the variables chosen to label settlements as *likely damaged* require further investigation and refinement. In the current implementation, the filtering relying on simple thresholding for VNF detections, frontline distances, and VNF ratios, excludes settlements with a low total number of emissions, omits strategic strikes away from the frontline, and requires a time series of equal length before and after an event. As such, near real-time detection of events is not yet possible. In spite of these limitations, the method offers a quick and flexible way to generate an overview of likely damaged settlements both spatially (for the entirety of Ukraine), and temporally (for the entirety of the war). The analysis of historical data could also aid in reconstruction efforts.

### Monitoring the state of Ukrainian heavy industry

**Fig. 4:**
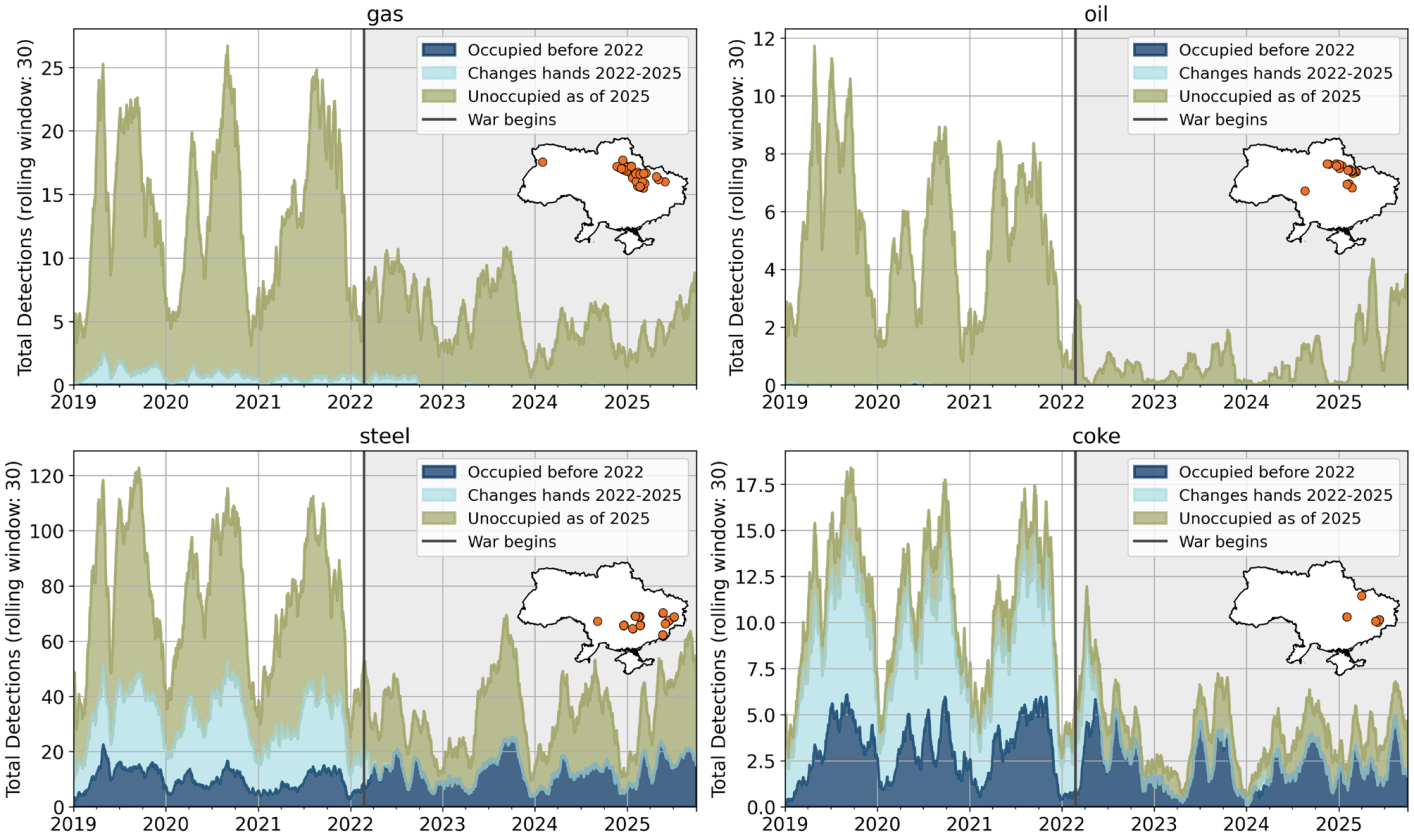
VNF fire detection time series for selected heavy industry types in Ukraine (30 day rolling window), divided by area of occupation. Emitter locations are shown in the inset map. All industries show a sharp drop in emissions with the outbreak of war, with different recovery rates per industry type. Please take note that *y* axes are scaled differently (data sources: VIIRS Nightfire, liveuamap.com).

Monitoring heavy industry using VIIRS thermal detections is not a novel idea^23–25^. However, by combining heavy industry location vector data^26^ with VNF detections and occupation data, several new insights can be generated on the state of heavy industry in Ukraine. Figure 4 shows the results of this approach, with VNF detections grouped by controlling entity. A 30-day rolling window was used to reduce short-term VNF variability and better reveal longer-term trends, which is appropriate for static industrial sites with continuous emissions while still capturing extreme events. Three insights become apparent. First, all heavy industry emissions in unoccupied Ukraine strongly decrease after the war begins. Second, all heavy industry in the areas occupied before 2022 continue to emit at the same intensity as before the war, even though many of the industry sites were less than 30 kilometres from the frontline when the invasion began. This suggests that these sites were never targeted in a concentrated effort by the Armed Forces of Ukraine. Third, all industry sites that are fought over stop emitting entirely, suggesting that industry sites that change hands have been damaged, and their operations cannot be easily restored.

**Fig. 5:**
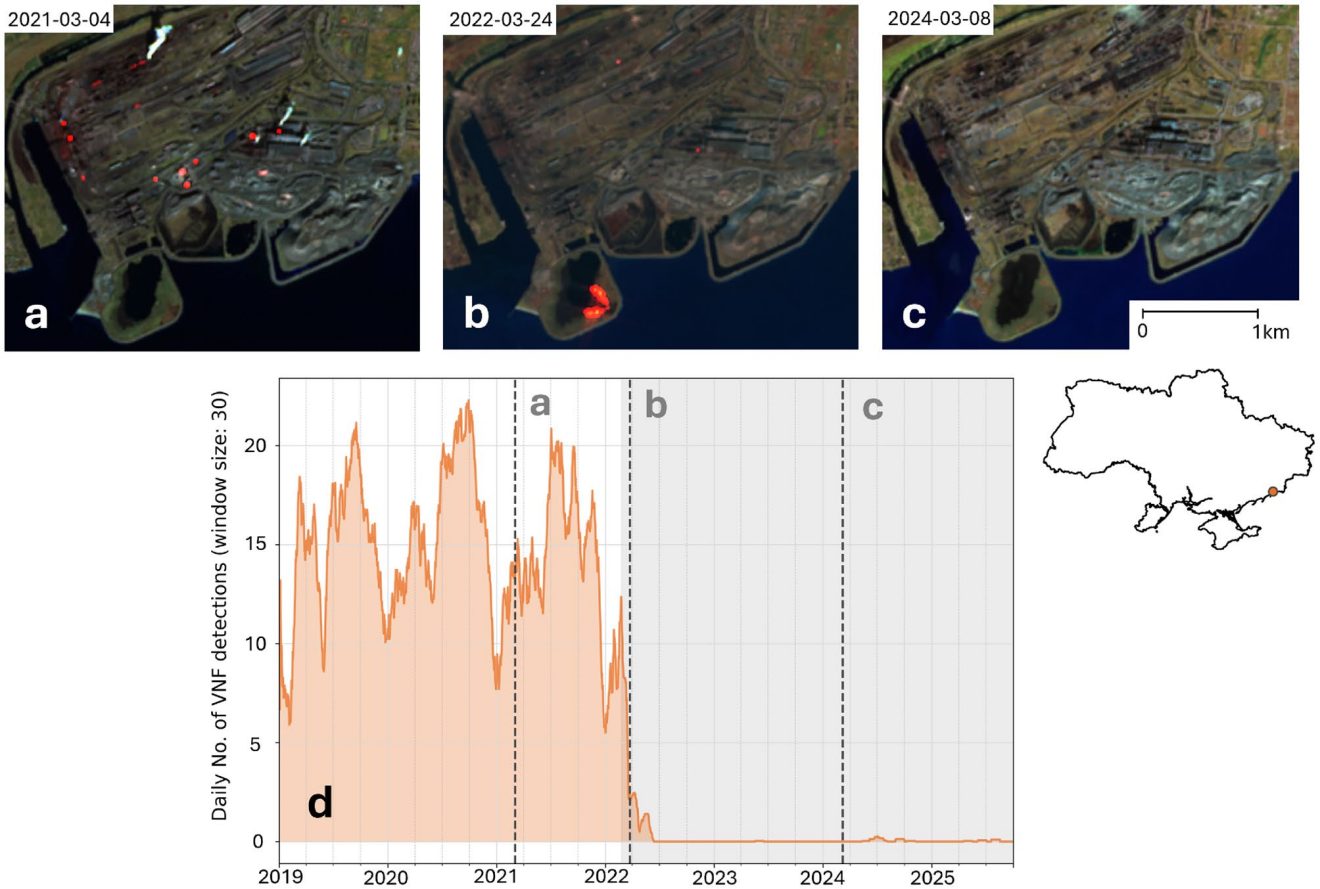
Cross analysis of Sentinel-2 SWIR false colour composites (**a**–**c**) and VNF detections (**d**) for the Azovstal steel plant in Mariupol. Sentinel-2 composites show SWIR emissions (highlighted in red) during regular operations (**a**), during active fighting (**b**) and after the city was captured (**c**). Data sources: Copernicus Space Data Ecosystem, VIIRS Nightfire.

Linking these results to actual industrial output is challenging due to limited data availability. However, a working paper from the United Nations Industrial Development Organization shows that all relevant Ukrainian manufacturing experienced a decline in production in 2022. Basic metals, coke, and refined petroleum saw a drop of over 60 percent in 2022 compared to the 2019–2021 average, with frontline regions suffering the most^27^. These trends are reflected in the VNF data. A second validation approach is the use of case studies and supplementary data, which was done for the large Azovstal steelworks in Mariupol. The city of Mariupol was one of the first to be attacked as the Russian invasion began due to its proximity to the pre-2022 frontline, and was the subject of heavy fighting. The Azovstal steelworks saw particularly intense combat, and were under siege for 80 days before being captured by the Russian Armed Forces^21^. This sequence of events is also visible when viewing VNF data and selected Sentinel-2 Short Wave Infrared (SWIR) composites (see Fig. 5).

Before the war began, the steelworks produced regular thermal emissions, which were distributed throughout the area. As Azovstal was attacked, all regular emissions ceased, and only sporadic emissions from active fires were visible. After the steelworks were captured, all emissions stopped and never restarted until the present day. Local photographs show the steelworks were completely destroyed^21^, with Russian officials reportedly planning to repurpose the site as a tourist destination^28^. The Azovstal case demonstrates how VNF data can be used to systematically monitor the functional status of industrial infrastructure throughout different phases of conflict.

### Towards frontline monitoring

During the four years of war, there has been a notable shift in the spatial pattern of VNF detections compared to the previous three years. It stands to reason that fighting around the frontline has an influence on the number and clustering of VNF detections, with fires caused by shelling and other explosions resulting in anomalies in thermal emissions. At the same time, the abandonment of agricultural lands^29^ might lead to a reduction in thermal emissions around areas of the frontline that are not subject to active combat, as controlled burning of fields no longer takes place. Other authors have demonstrated initial approaches for assessing the relationship between fighting in Ukraine and patterns of fire detection^16,17^. However, the relationship between frontline and VNF distribution is challenging to investigate, as the former constantly moves, as does the disposition of firepower. However, by combining the weekly frontline data set with VNF locations, it becomes possible to calculate the distance of each VNF detection from the frontline *at the time it was detected*.

**Fig. 6:**
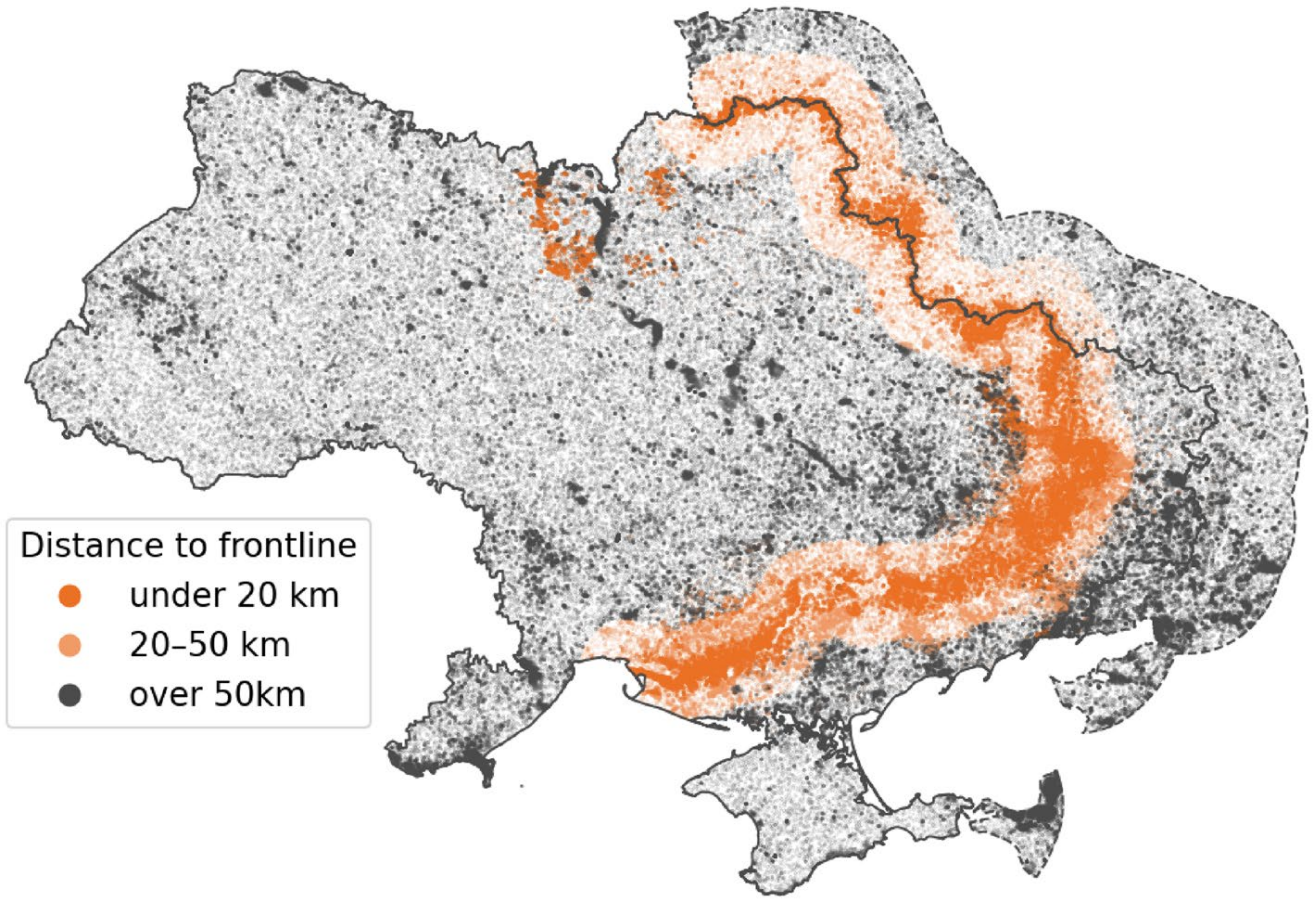
Composite of VNF detections during the entire war (2022-02-24 to 2025-09-30) by distance from the frontline in Ukraine and a 150km buffer of the Russian border. It should be noted that the large number of VNF detections near the Dnipro river and Pripyat marshes are likely artifacts caused by land-water temperature contrasts (data sources: OCHA, VIIRS Nightfire, liveuamap.com).

The results of this approach can be seen in Fig. 6, which shows an aggregated map of all VNF detections during the entire war, with the respective distances to the frontline and area changes. The large “belt” of detections that appeared after the war began are close to the frontline, with most detections in the Donbas, Kherson, Kharkiv and Kyiv regions occurring less than 20 kilometres from the frontline. When grouping Ukrainian VNF detections by *urban* and *non-urban* using settlement boundary data, it also becomes apparent that the distribution of urban VNF detections is grouped much closer to the frontline than non-urban ones. However, it is also clear that events related to warfare are not the only cause of VNF anomalies, and the question remains, if a temporal resolution higher than the three-year composites shown here would also show clear patterns.

To further investigate these questions, the VNF data were aggregated by month into 5 $\textrm{km}^{2}$ grid cells, after which global and local Moran’s I was calculated. Moran’s I is a measure of spatial autocorrelation, showing whether a feature (in this case a grid cell) is surrounded by features with similar values^30^. Values range from −1 to +1, where higher values indicate that similar patterns occur close to one another, and lower values indicate more scattered patterns. At the global level, Moran’s I shows whether VNF anomalies tend to cluster across the study area as a whole. At the local level, it identifies where such clustering is strongest, highlighting areas where elevated or reduced VNF activity occurs together with similar activity in neighbouring grid cells (*high-high* and *low-low* clusters). These clusters represent spatial concentrations of change, which can serve as an indicator of sustained disruption and, by extension, the location of the frontline.

**Fig. 7:**
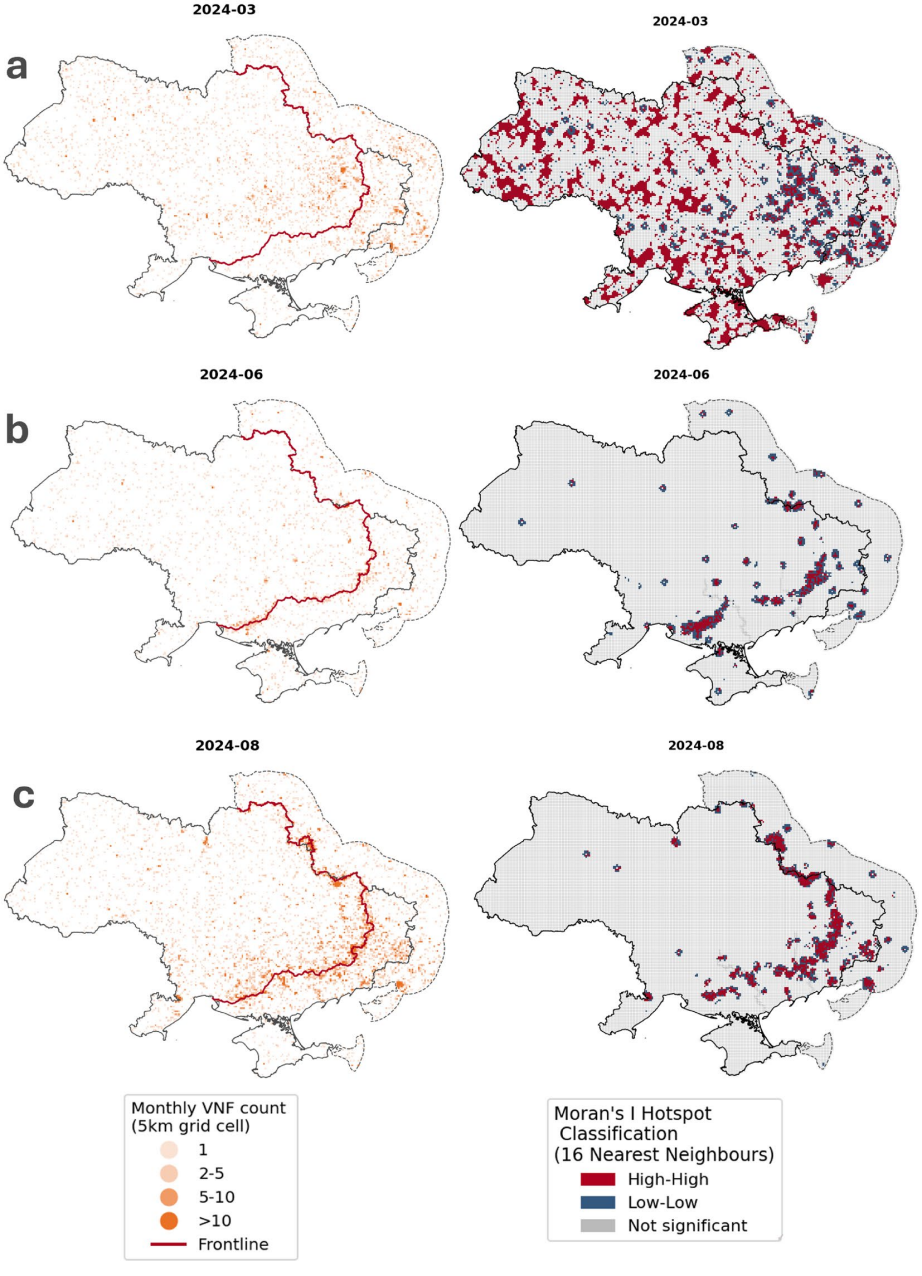
Examples of poor (**a**), moderate (**b**) and good (**c**) matches between the frontline location, VNF detections, and Moran’s I hot spots per month. VNF detections are aggregated by month in 5km grid cells. (data sources: OCHA, VIIRS Nightfire, liveuamap.com).

The results show that the global average monthly spatial autocorrelation for VNF detections ranges from very weak (Moran’s I of 0.05) to moderate (Moran’s I of 0.25) when considering the 16 nearest neighbours. There are large deviations per month, with higher autocorrelation values occurring in months with a larger number of VNF detections. There are only small deviations in autocorrelation before and after the war begins, with autocorrelation being higher during August and September than before 2022, which could point to the war influencing the spatial distribution of VNF detections. However, it should be kept in mind that only six years in total are being observed, and the summer months have lower cloud coverage, limiting the significance of this occurrence.

On a local scale, autocorrelation is very heterogeneous, with strong variations per month and per season. Figure 7 shows three examples of this variation, with poor, moderate and good matches between VNF detections, Moran’s I hot spots and frontline location. During winter months and in late spring, the low number of total VNF detections leads to large clusters of *high-high* grid cells in areas without any detections, while the number of detections around the frontline is also much lower. This combination of occurrences makes it impossible to spot the frontline from autocorrelation data. However, during late spring to late summer, the clustering of hot spots is more significant. Here, hot spots are arranged as a swath of *high-high* hot spots, surrounded by a ring of *low-low* hot spots, caused by the sharp drop in the number of VNF detections with increasing distance from the frontline. This seasonality in frontline detectability is likely caused by the interplay of weather patterns and combat intensity: during winter months, cloud cover is highest^31^, limiting the number of detections. The wet and muddy seasons of fall and especially spring make mechanized advances in Ukraine very difficult^32^, limiting combat intensity but also decreasing the likelihood of fires starting.

Compared to previous insights, frontline monitoring comes with several caveats: first, the frontline is calculated only on a weekly or monthly basis, meaning that the actual frontline might deviate from the calculated version. This is especially the case during the first two months of the war, when fighting was much more dynamic. Second, the frontline is calculated using a buffered overlay of occupied areas, which can be imprecise and leave frontline gaps or false area changes. Third, while both linear and geographically weighted regression models were tested, no statistically significant linear relationship between the distance from the frontline and the number of VNF detections could be established. As such, further research into separating *frontline* VNF detections from *background* VNF detections should be conducted. An additional challenge is posed by events along sections of the frontline not generating thermal anomalies detectable by VIIRS. Still, with improved methods and better supporting data such as land cover labels or geolocated web event data, frontline monitoring using VNF detections has the potential to become a useful tool for tracking conflicts.

## Discussion

### Data limits

VIIRS is a sensor characterized by a very high temporal and a very low spatial resolution. However, both the thermal and visible radiances are subject to very strong fluctuations, limiting the interpretability of individual images. For VIIRS DNB, the main sources of uncertainty are angular and atmospheric effects^33^. Additionally, cloud cover can either darken or brighten the sky depending on the amount of artificial light present^2^, with even monthly composites showing radiances that are 10-30 percent lower than the average when the number of cloud-free daily images is low^34^. While VIIRS’ thermal bands can register emissions through limited cloud cover, they are still subject to strong fluctuations. On a practical level, the likelihood of detecting a fire caused by conflict-related damage is relatively low, as several events need to happen concurrently for it to be registered. First, the average temperature of the target pixel must generate SWIR radiation above the detection threshold. Second, the anomaly in temperature must be persistent and concurrent with the satellite overpass. Finally, the target must not be obscured by cloud cover.

These limits have two consequences. First, short-time events such as airstrikes or temporary power outages might not be visible, except in specific cases. As VIIRS captures all the data in a four-hour nighttime window, even fleeting cloud cover can stop a detection from occurring. As an example, the Russian Tuapse oil refinery was attacked twice by Ukrainian drones with subsequent fires, which were not detected by VIIRS. It should be noted that this temporal distribution is not as problematic when looking at static targets, where nighttime is preferred due to smaller detection limits against the sensor noise floor^26^. Second, as average cloud cover in Ukraine fluctuates by season with over 80 percent in December and under 50 percent in August^31^, the likelihood of an emission being detected by VIIRS also fluctuates, resulting in a relevant number of false negatives.

Another limitation is the the role of external events such as forest fires and crop burning, as well as the influence of land cover in general. As seen in Fig. 6, large numbers of VNF detections occur far away from the front line. As 47 percent of Ukraine consists of cultivated land, and 70 percent of total land coverage is dedicated to agricultural use, the burning of crop residues, as often practised in Ukraine^35^, is likely to have a large effect on the number of VNF detections. Additionally, Ukraine saw an extreme increase in wildfire detections in 2024 due to a high Fire Weather Index, comprising half of the total mapped burnt area in the entire region covered by the European Forest Fire Information System (Europe and parts of North Aftrica and parts of the Middle East)^36^. Given the proximity to the frontline, it is likely that many of these detections are caused by military activity, but it cannot be conclusively proven. As both the burning of crops and the likelihood of natural forest fires are highly seasonal, further investigations into the seasonality of VNF detections by land cover type^16^ would be helpful, and could also help remove detection errors over swamps and lakes.

A last point of discussion is that even if an area of interest shows a sustained drop or increase in emissions, the data must be averaged over a time window to establish if the observed fluctuation is a real change rather than noise. As a result, the temporal resolution of observable events decreases. Extreme shifts in emissions such as those in large destroyed industrial sites like Azovstal, might be visible within a single night (barring cloud cover), but smaller areas of interest with more nuanced changes are more difficult to detect initially. In this study, VNF detections were temporally smoothed using a simple rolling window approach, but other methods might be available that are better suited. As such, the following recommendations can be made to increase method robustness: Cloud cover should be further integrated into any VIIRS monitoring system, with further investigations into the interplay of seasons, weather conditions, land cover, and the likelihood of damage detections. Lastly, the minimum required temporal averaging should be investigated: the smaller the time window used, the quicker a change in emissions can be classified as substantial, efficiently leveraging the high temporal resolution VIIRS can offer.

#### On the use of multispectral rasters

In this study, an approach based on a derivative VIIRS data set (VNF) was chosen instead of relying on processed VIIRS multiband raster data that is also freely available. There are several reasons for this choice: point-based vector data can be easily manipulated and combined with other information layers, and the amount of data that needs to be processed is substantially lower. Additionally, the data are simple to interpret, as multi-band rasters are transformed into geolocated *fire detections*. In much of the literature, VIIRS thermal data in conflict monitoring are derivative based, mainly using FIRMS^16,17^. In contrast, VIIRS visible night light use is mainly raster based^8–10^. The potential advantage of directly using raster data is that more usable information such as multiband detections, temperature calculations and cloud cover presence are available for each image element, allowing taking into account the context of each detection.

## Conclusion

This study showed how VNF can be integrated with boundary data, territorial control vector data and UNOSAT damage assessment maps to generate a series of spatial and temporal conflict indicators. These indicators were then used to generate insights on heavy industry status, locations of urban combat, and frontline movements across Ukraine. Results show that especially the first two applications show high potential to be a simple and reliable tool for monitoring large, conventional conflicts.

The war in Ukraine stands out as one of the largest peer-to-peer conflicts in recent history. The results from this study show that VIIRS data, despite its limitations, can provide meaningful insights into conflict dynamics when integrated with supporting data. While transferability to other conflicts remains to be assessed, the war in Ukraine constitutes a particularly suitable case for analysis due to both the intensity of the fighting and the availability of supporting geospatial data. Many conflicts, particularly in the Middle East and Africa, lack one or both of these characteristics, which may limit direct transferability. Nevertheless, an initial test on the Gaza Strip indicates that transferability may be feasible with appropriate adjustments. When further validated and supported by complementary data, VNF and VIIRS thermal data more broadly could thus become a powerful tool for independent conflict monitoring. In an unstable world, such capabilities are critical for a timely and impartial understanding of events.

## Methods

**Fig. 8:**
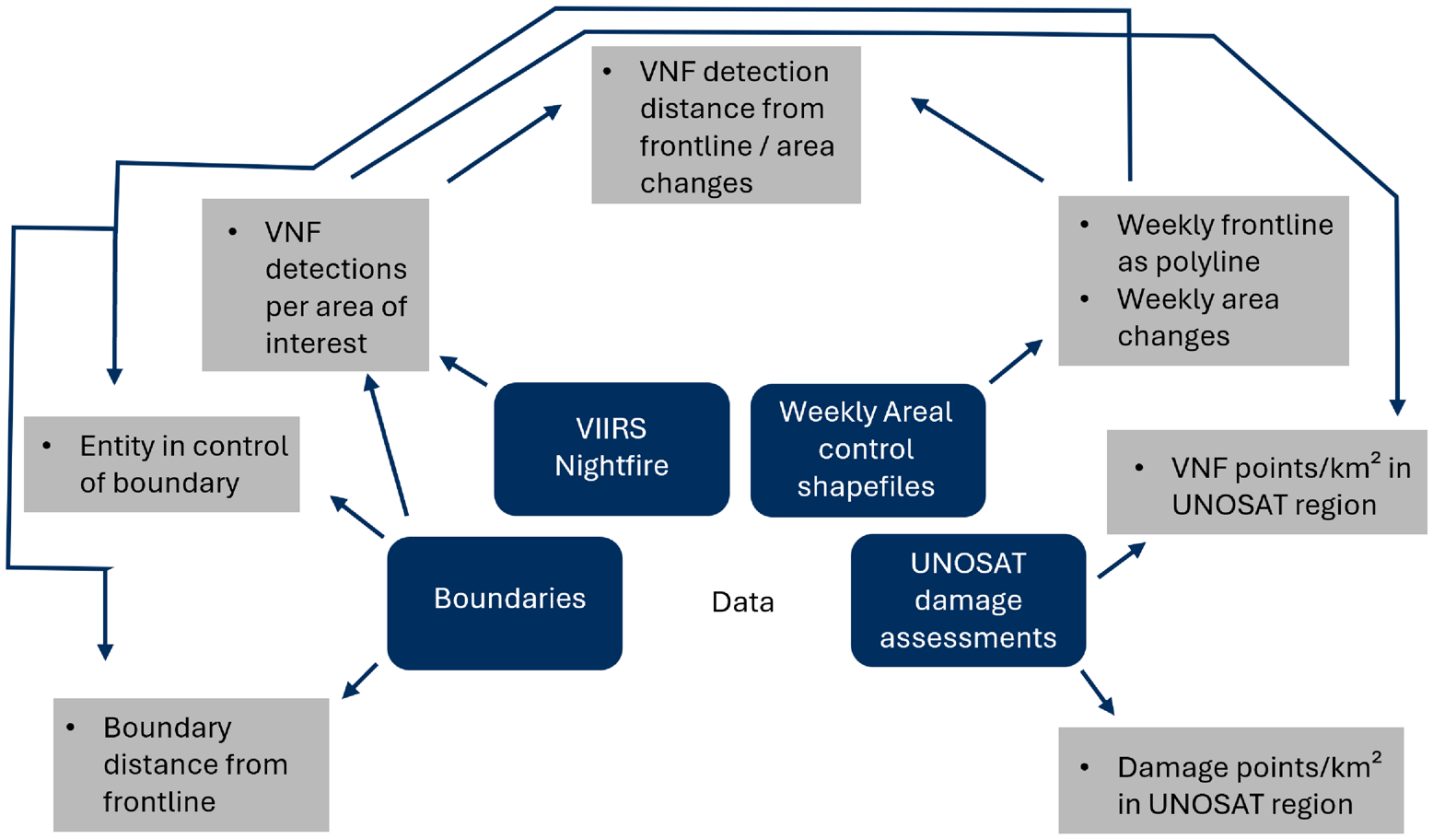
Generation of indicators for conflict monitoring. Data products are highlighted in blue, generated indicators are shown in grey. All results in this study are produced using a combination of these indicators.

As this study consists of several use cases, there is no single methodology. Instead, this chapter will describe how VIIRS data and other geospatial data were shaped to generate key indicators on the war in Ukraine. A broad overview can be seen in Fig. 8. Except for area control data, all data used are either entirely open-source, or provided free of charge through non-commercial or academic licenses. Additionally, all data were processed with minimal supervision, allowing for easy transferability to other regions and time periods.

### VIIRS Nightfire

The dataset chosen for monitoring thermal emissions is VIIRS Nightfire (VNF) version 3.0^3–5^. VNF detects hot sources at sub-pixel level using a combination of spectral bands. In VNF, heat sources are independently detected using two distinct detectors: the first uses thresholding on the M10 and M11 SWIR bands, while the second uses a M12–M13 MWIR band decorrelation technique^37^. Central wavelengths are 1601 nm for M10, 2250 nm for M11, 3730 nm for M12 and 4066 nm for M13. While the detectors are independent, several bands require a signal above the noise level in order to calculate temperature and source area estimates. These estimates are calculated by applying spectral unmixing between the heat source and background Planck curves^38^ (i.e. how much radiation is emitted at each wavelength for a given temperature for a black body), detecting hot sources from 600 to 6000 K. A pixel is considered *hot* if its band radiance exceeds the mean plus four standard deviations of background pixels, calculated separately for three pixel aggregation zones. The radiance of corresponding bands is then checked using a similar procedure. If a detection is only found in one of the SWIR bands but not in any other band, it is marked as *unconfirmed*, which either means the signal was weak or the detection was a result of noise. The test data set used by Elvidge et al.^3^ contained about 44 percent unconfirmed detections. VNF sensitivity is dependent on the heat source temperature, but is estimated to range between $<{\textrm{1 m}}^{2}$ for gas flares and $>10\,{\textrm{m}}^{2}$ for forest fires^4^.

VNF was originally designed to monitor gas flares at night^39^, but records and stores all thermal anomalies. While a newer version of VNF exists which includes secondary sub-pixel emissions^5^ this study uses V3.0 for analysis. This decision was taken as V3.0 contains a homogenous timeseries from 2017 until the present day, which allows for the establishment of a baseline of fire detections before the outbreak of hostilities. For the same reason, data from NOAA-21 was excluded from the analysis, as data from this platform became available only in 2023. VNF merges several VIIRS detections per night. For processing, all data from 2019-01-01 to 2025-09-30 were transformed into point vector layers and subsetted to the extent of Ukraine, the Black Sea, and areas of Russia within a 100 km range of Ukraine. Lastly, all vector data were merged, allowing for easy sorting and subsetted to specific areas of interest in following operations. This merged vector file formed the basis of all further VNF operations.

For detecting the frontline using VNF detections, the data was grouped by month into 5x5 km grid cells, after which the statistical autocorrelation method of Moran’s I was used, according to the following equation:

1 $$\begin{aligned} I = \frac{N}{W} \frac{\sum _{i=1}^{N} \sum _{j=1}^{N} w_{ij} (x_i - \bar{x})(x_j - \bar{x})}{\sum _{i=1}^{N} (x_i - \bar{x})^2} \end{aligned}$$

where *N *is the number of spatial units indexed by *i* and *j*, $\textit{x}_\textit{i}$ is the value of the variable at location *i*, $\bar{x}$ is the mean of the variable *x*, $\textit{w}_\textit{ij}$ is the spatial weight between units *i* and *j*, and *W* is the sum of all spatial weights. Values range from −1 (strong negative autocorrelation) to +1 (strong positive autocorrelation). At a global level, Moran’s I provides insights into the spatial autocorrelation of the entire dataset. At a local level, it enables the identification of specific hot spots characterized by high or low autocorrelation. Here, *high-high *clusters consist of grid cells with high autocorrelation surrounded by grid cells that also show high autocorrelation, while *low-low* clusters consist of grid cells of low autocorrelation, surrounded by other low-autocorrelation grid cells.

### Boundary data

To automatically divide visible and thermal emissions by area of interest, boundary data were required. For this task, several datasets were used (see Table 1): data on settlement outlines and regional boundaries were taken from the UN’s OCHA office, and permanent thermal emitters were taken from VNF-generated permanent emitter vector data. An indicator of *daily number of VNF detections in boundary* was derived by combining the boundaries with relevant VNF detections.

**Table 1:** Boundary data used.

Dataset	Information	Source
Ukrainian settlement boundaries	Boundaries of every settlement in Ukraine	Data.humdata.org
Russian administrative boundaries	Administrative boundaries of Russia’s Kursk oblast	Data.humdata.org
Permanent thermal emitters	Locations of heavy industry with thermal emissions (worldwide)	Colorado School of Mines
Port boundaries	Boundaries of selected Ukrainian and Russian ports	Manually drawn from ArcGIS Pro basemap imagery

### Occupied areas

One feature of modern conflicts is that it is possible for non-state actors to assess areas of control through a variety of data, such as official statements, geolocated images, and social media data. There are several organizations that provide areas of control data in vector format^18,40–42^. The data used in this project comes from *Liveuamap*, an NGO that provides constant updates on several conflict regions around the world, with a focus on the war in Ukraine^18^. The organization offers daily data in vector format and notable events on the war in Ukraine through an online subscription service.

As the data needed to be downloaded manually, these were selected only once per week. First, areas occupied before 2022 were removed. Second, non-area geometries were removed, and small geometries remaining from clipping operations were deleted. Third, territorial changes and their size were extracted through several subsetting operations in Ukraine and the Russian regions bordering Ukraine. For each territorial change, the area was calculated. If data were missing, they were manually replaced with data from the previous week. These steps were repeated from the beginning of the war until 2025-09-30, netting the indicators of *weekly occupied area*, *weekly territorial changes,* and *weekly frontline as polyline*. By combining these outputs with the boundary and VNF data, it was also possible to generate the indicators of *distance of settlement from frontline*/*territorial change*, *distance of VNF detection from frontline/territorial change *and *number of VNF emissions by occupied/unoccupied area*.

### UNOSAT damage assessment data

The United Nations Satellite Centre (UNOSAT) has a team of analysts delivering damage maps of crisis areas (mainly settlements) relying on VHR optical imagery^19^. Their output includes damage assessments of several Ukrainian towns and cities, mainly during the opening stages of the war. The programme works by comparing VHR-imagery before and after a damage event using visual analysis and manual annotation of damaged buildings and infrastructure. The *Comprehensive Damage Assessment* (CDA) data product from UNOSAT provides a map with damage locations and damaged objects as points in vector format.

For this study, CDA data were downloaded for 14 Ukrainian settlements (15 total activations), which are all CDA activations in Ukraine not related to flooding. Since the boundaries of the analysed areas were only provided in non-georeferenced map form, they were manually converted to vectors. If the damage assessment used more than one post-event image, the latest date was selected. The area of each region was then calculated to derive the indicators of *damaged buildings per area* and *VNF detections per area* for each UNOSAT activation between the beginning of the war and the latest post-event image, enabling cross-analysis.

### Visual validation data

In addition to the data products listed so far, Sentinel-2 (S-2) optical data was used *as is* for visual validation^43^. S-2 offers a spatial resolution of up to ten meters and a temporal resolution of approximately five days, depending on latitude. For verifying VNF fire detections, S-2 composites using the B12 (SWIR), B8 (near-infrared) and B4 (red) bands were used, as this band composite highlights thermal emissions resulting from high temperatures. The composites were resampled to 20 meters, matching the resolution of the B12 SWIR band. Due to cloud cover, relevant S-2 images were only available in limited number. In future, S-2 data could be further integrated into a detection framework, but for now, visual identification and validation is adequate for the study design chosen. It should be kept in mind that VNF detections are geolocated at the VIIRS pixel center, which has a footprint of 750x750 meters, and hot sources could be located anywhere within an area of this size.

## Data Availability

Due to data licensing restrictions and the coverage of an ongoing conflict, not all data can be freely redistributed. Data availability is as follows: Settlement and administrative boundary data is freely available from OCHA’s website (see Table 1). UNOSAT damage assessment data is freely availably from UNOSAT’s website at https://unosat.org/products/. Permanent thermal emitter boundaries and VIIRS Nightfire data was used under an academic license from the Colorado School of mines, which does not permit redistribution in any form. However, the data can be licensed free of charge for academic purposes at the Colorado school of mines at https://eogdata.mines.edu/products/vnf/. Occupied area data were extracted using a paid subscription service from liveuamap.com. The data can be downloaded at https://liveuamap.com with a subscription. The code used for data preparation and analysis is available upon reasonable request to the authors.

## References

[1] Jójárt, K. The war against Ukraine through the prism of Russian military thought. *J. Strateg. Stud.* **47**, 801–831. 10.1080/01402390.2024.2414079 (2024).

[2] Levin, N. et al. Remote sensing of night lights: A review and an outlook for the future. *Remote Sens. Environ.* **237**, 111443. 10.1016/j.rse.2019.111443 (2020).

[3] Elvidge, C., Zhizhin, M., Hsu, F.-C. & Baugh, K. Viirs Nightfire: Satellite pyrometry at night. *Remote Sens.* **5**, 4423–4449. 10.3390/rs5094423 (2013).

[4] Elvidge, C. D., Zhizhin, M., Baugh, K., Hsu, F. C. & Ghosh, T. Extending nighttime combustion source detection limits with short wavelength viirs data. *Remote Sens.* **11**, 395. 10.3390/rs11040395 (2019).

[5] Elvidge, C. D., Zhizhin, M., Ghosh, T., Hsu, F.-C. & Taneja, J. Annual time series of global viirs nighttime lights derived from monthly averages: 2012 to 2019. *Remote Sens.* **13**, 922. 10.3390/rs13050922 (2021).

[6] Li, X., Zhang, R., Huang, C. & Li, D. Detecting 2014 northern Iraq insurgency using night-time light imagery. *Int. J. Remote Sens.* **36**, 3446–3458. 10.1080/01431161.2015.1059968 (2015).

[7] Li, X., Liu, S., Jendryke, M., Li, D. & Wu, C. Night-time light dynamics during the Iraqi civil war. *Remote Sens.* **10**, 858. 10.3390/rs10060858 (2018).

[8] Li, L.-L., Liang, P., Jiang, S. & Chen, Z.-Q. Multi-scale dynamic analysis of the Russian–Ukrainian conflict from the perspective of night-time lights. *Appl. Sci.* **12**, 12998. 10.3390/app122412998 (2022).

[9] Chen, M., zhang, H., Hu, S., Zhoufang, X. & Tian, D. Nighttime satellite light view of Kherson on the front line of the Russia–Ukraine conflict. In Shao, X. (ed.) *Third International Computing Imaging Conference (CITA 2023)*, 55 (SPIE, 2023). 10.1117/12.2688194

[10] Xiao, B. et al. Night-time light loss during the 2022 kyiv offensive revealed through viirs dnb. *Eur. J. Remote Sens.*10.1080/22797254.2024.2362387 (2024).

[11] Xu, H., Barbot, S. & Wang, T. Remote sensing through the fog of war: Infrastructure damage and environmental change during the Russian–Ukrainian conflict revealed by open-access data. *Nat. Hazards Res.* **4**, 1–7. 10.1016/j.nhres.2024.01.006 (2024).

[12] Cerra, D., Merkle, N., Henry, C., Gapp, S. & Gstaiger, V. Increases in night lights intensity reveal extreme events: A case of study on the ongoing conflict in Ukraine. ISPRS Annals of the Photogrammetry, Remote Sensing and Spatial Information Sciences X-3-2024, 53–59 (2024). 10.5194/isprs-annals-X-3-2024-53-2024.

[13] Schroeder, W., Giglio, L., Hall, J. & VIIRS 375-m Active Fire Product User’s Guide Version 1.0. Collection 2 Visible Infrared Imaging Radiometer Suite (VIIRS) 375-m Active Fire Product User’s Guide Version 1.0 (2024).

[14] Ialongo, I., Bun, R., Hakkarainen, J., Virta, H. & Oda, T. Satellites capture socioeconomic disruptions during the 2022 full-scale war in Ukraine. *Sci. Rep.* **13**, 14954. 10.1038/s41598-023-42118-w (2023). 37737292 10.1038/s41598-023-42118-w PMC10516891

[15] Yailymov, B., Yailymova, H., Shelestov, A. & Shumilo, L. Monitoring of fires caused by war in Ukraine based on satellite data. In *2023 13th International Conference on Dependable Systems, Services and Technologies (DESSERT)*, 1–5 (IEEE, 2023). 10.1109/dessert61349.2023.10416520

[16] Tomchenko, O. V. et al. Assessment and monitoring of fires caused by the war in Ukraine on landscape scale. *J. Landsc. Ecol.* **16**, 76–97. 10.2478/jlecol-2023-0011 (2023).

[17] Naghizadeh, M. H. The uses for fire data and satellite images in monitoring, detecting, and documenting collective political violence. *Res. Politics*10.1177/20531680241261769 (2004).

[18] Liveuamap.com. Ukraine interactive map—Ukraine latest news on live map (2025). Available at: https://liveuamap.com/.

[19] UNOSAT. Unosat activation list (2025). Available at: https://unosat.org/products/.

[20] Brands, H. *War in Ukraine: Conflict, Strategy, and the Return of a Fractured World* (Johns Hopkins University Press, 2024).

[21] Schwirtz, M. Last stand at Azovstal: Inside the siege that shaped the Ukraine war. The New York Times 2022 (2022).

[22] Bugayova, N. Ukraine’s sustained counteroffensive: Denying Russia’s prolongation of the war (2023). Available at: https://www.understandingwar.org/backgrounder/ukraine.

[23] Liu, Y. et al. Identifying industrial heat sources using time-series of the viirs nightfire product with an object-oriented approach. *Remote Sens. Environ.* **204**, 347–365. 10.1016/j.rse.2017.10.019 (2018).

[24] Ma, C. et al. Assessing the distribution of heavy industrial heat sources in India between 2012 and 2018. *ISPRS Int. J. Geo Inf.* **8**, 568. 10.3390/ijgi8120568 (2019).

[25] Sun, S. et al. Variation of industrial air pollution emissions based on viirs thermal anomaly data. *Atmos. Res.* **244**, 105021. 10.1016/j.atmosres.2020.105021 (2020).

[26] Elvidge, C. D. et al. Global satellite monitoring of exothermic industrial activity via infrared emissions. *Remote Sens.* **15**, 4760. 10.3390/rs15194760 (2023).

[27] UNIDO. The impact of the war on industrial sectors in Ukraine. Inclusive and Sustainable Industrial Development Working Paper Series (2024).

[28] Vladislava. Russian officials to turn azovstal steel works into a tourist attraction. bukvy 2024 (2024).

[29] Mkrtchian, A. & Müller, D. Assessing the impact of the Russian invasion on crop production in Ukraine with open satellite data. *Ukr. Anal. Digest*10.3929/ETHZ-B-000665476 (2024).

[30] Giungato, G. & Maggio, S. Moran’s index. Encyclopedia of Mathematical Geosciences 897–901 (2023). 10.1007/978-3-030-85040-1_210.

[31] Osborn, T. J., Wallace, C. J., Harris, I. C. & Melvin, T. M. Pattern scaling using climgen: Monthly-resolution future climate scenarios including changes in the variability of precipitation. *Clim. Change* **134**, 353–369. 10.1007/s10584-015-1509-9 (2016).

[32] Kagan, F. W. Russian offensive campaign assessment, December 30, 2023 (2022). Accessed 29 Jan 2026. Available at: https://www.understandingwar.org/backgrounder/russian-offensive-campaign-assessment-december-30-2023.

[33] Wang, Z. et al. Quantifying uncertainties in nighttime light retrievals from suomi-npp and noaa-20 viirs day/night band data. *Remote Sens. Environ.* **263**, 112557. 10.1016/j.rse.2021.112557 (2021).

[34] Patnaik, A., Shah, A., Tayal, A. & Thomas, S. But clouds got in my way: Bias and bias correction of viirs nighttime lights data in the presence of clouds. *SSRN Electron. J.*10.2139/ssrn.3957319 (2021).

[35] Hall, J. V. et al. Environmental and political implications of underestimated cropland burning in Ukraine. *Environ. Res. Lett.* **16**, 064019. 10.1088/1748-9326/abfc04 (2021). 34316296 10.1088/1748-9326/abfc04 PMC8312694

[36] San-Miguel-Ayanz, J. et al. Advance Report on Forest Fires in Europe, *Middle East and North Africa 2024*. JRC141505 (Luxembourg, 2025).

[37] Zhizhin, M., Elvidge, C. & Poyda, A. Night-time detection of subpixel emitters with viirs mid-wave infrared bands m12–m13. *Remote Sens.* **15**, 1189. 10.3390/rs15051189 (2023).

[38] Planck, M. On the law of the energy distribution in the normal spectrum. *Ann. Phys.* **4**, 1–11 (1901).

[39] Zhizhin, M. et al. Measuring gas flaring in Russia with multispectral viirs nightfire. *Remote Sens.* **13**, 3078. 10.3390/rs13163078 (2021).

[40] ISW. Isw Ukraine invasion interactive web map (2025). Available at: https://www.arcgis.com/home/item.html?id=9f04944a2fe84edab9da31750c2b15eb.

[41] Geoconfirmed. Ukraine - geoconfirmed (2025). Available at: https://geoconfirmed.azurewebsites.net/ukraine/.

[42] Project Owl. Ukraine control map v2 (2025). Available at: https://uacontrolmap.com/.

[43] Drusch, M. et al. Sentinel-2: Esa’s optical high-resolution mission for gmes operational services. *Remote Sens. Environ.* **120**, 25–36. 10.1016/j.rse.2011.11.026 (2012).

